# Shortening a Patient Experiences Survey for Medical Homes

**DOI:** 10.3390/healthcare4010001

**Published:** 2015-12-23

**Authors:** Judy H. Ng, Erika Henry, Tyler Oberlander, Peichang Shi, Sarah Hudson Scholle

**Affiliations:** 1 National Committee for Quality Assurance, 1100 13th Street NW, Suite 1000, Washington, DC 20005, USA; ehenry@ncqa.org (E.H.); oberlander@ncqa.org (T.O.); scholle@ncqa.org (S.H.S.); 2 Booz Allen Hamilton, One Preserve Parkway, Suite 200, Rockville, MD 20852, USA; shi_peichang@bah.com

**Keywords:** Patient-Centered Medical Homes, PCMH, patient reports about care, health care quality, patient experiences survey, CAHPS^®^

## Abstract

The Consumer Assessment of Healthcare Providers and Systems—Patient-Centered Medical Home (CAHPS PCMH) Survey assesses patient experiences reflecting domains of care related to general patient experience (access to care, communication with providers, office staff interaction, provider rating) and PCMH-specific aspects of patient care (comprehensiveness of care, self-management support, shared decision making). The current work compares psychometric properties of the current survey and a proposed shortened version of the survey (from 52 to 26 adult survey items, from 66 to 31 child survey items). The revisions were based on initial psychometric analysis and stakeholder input regarding survey length concerns. A total of 268 practices voluntarily submitted adult surveys and 58 submitted child survey data to the National Committee for Quality Assurance in 2013. Mean unadjusted scores, practice-level item and composite reliability, and item-to-scale correlations were calculated. Results show that the shorter adult survey has lower reliability, but still it still meets general definitions of a sound survey for the adult version, and resulted in few changes to mean scores. The impact was more problematic for the pediatric version. Further testing is needed to investigate approaches to improving survey response and the relevance of survey items in informing quality improvement.

## 1. Introduction

The Patient-Centered Medical Home (PCMH) care model is gaining prominence as a way of improving primary care. The PCMH is commonly defined by an emphasis on comprehensive, team-based care; patient-centered care; coordination across different aspects of the health care system; access to care; and a commitment to quality and safety [1]. The adoption of PCMH functions have been encouraged under the Affordable Care Act, and multiple payers have provided financial incentives for practices to become medical homes [2,3].

As PCMH adoption expands, the ability to evaluate patient experiences has become critical in evaluating the impact of the medical home [4]. With Commonwealth Fund support, NCQA collaborated with the Consumer Assessment of Healthcare Providers and Systems (CAHPS^®^, Agency for Healthcare Research and Quality, Rockville, MD, USA) Consortium, overseen by the Agency for Healthcare Research and Quality (AHRQ), to develop a survey instrument functionally aligned with key PCMH functions. This instrument, the CAHPS PCMH, finalized in late 2011, evaluates patient experiences on key domains of care associated with the medical home [5], and has since been used in a variety of settings, including National Committee for Quality Assurance’s (NCQA) PCMH recognition program [6]. A few studies have provided some support for the reliability and validity of the survey [3,7], though their results were based on initial data or smaller samples than those used in our current study (almost 7500 respondents in two census regions across prior studies [3,7]).

Nonetheless, even as several national and state initiatives have adopted the CAHPS PCMH version for public reporting or evaluation efforts [8,9], there have been concerns raised about the length of the survey. For example, low uptake rates have persisted since the survey was first introduced for use in the NCQA’s PCMH recognition program, the most widely used method for qualifying practices for rewards in multi-payer PCMH demonstrations [3,5]. While over 11,000 practices, representing an estimated 15%–18% of primary care physicians, are currently recognized by NCQA [10], fewer than 3% of them submit patient experiences surveys to NCQ when applying for recognition under NCQA’s PCMH recognition program.

Based on these concerns, and growing attention to survey length, NCQA evaluated and shared initial psychometric results, and gathered qualitative input from multiple stakeholders to make recommendations for shortening the survey used for NCQA’s programs. Stakeholders included about a dozen clinicians, researchers, survey implementers, those who work with practices to improve patient experiences, and those who use the survey for public reporting purposes, as well as about a dozen patient advocates and a separate broad-based advisory panel.

The growing attention to survey length is not limited to NCQA efforts, and attempts to address this concern have also grown. For example, in the months since NCQA initiated its evaluation, the CAHPS Consortium also released a slightly shorter version of the CAHPS Clinician and Group survey (version 3.0), which forms the core of the CAHPS PCMH survey, reducing the length from 34 to 31 items [11,12]. Additional possibilities for shortening the CAHPS Clinician and Group survey were also published recently [13].

This paper compares the psychometric properties of the original and proposed shortened survey based on the CAHPS PCMH survey. We used data reported in 2013 by 326 practices (including over 30,000 patients from three census regions), using data reported by medical practices in 2013. The proposed reductions shorten the adult survey from 52 to 26 items (from 10 to 5 pages), and the child survey from 66 to 31 items (from 12 to 6 pages).

## 2. Methods

### 2.1. Data and Materials

Data were from practices that voluntarily submit CAHPS PCMH data to NCQA as part of NCQA’s PCMH recognition program. All practices used NCQA-certified survey vendors to collect data. NCQA requires all survey vendors participate in annual training and monitoring of survey administration procedures.

Practices may submit data on the adult or child versions of the CAHPS PCMH survey items. The CAHPS PCMH survey uses the CAHPS Clinician and Group (C&G) core survey (version 2.0), plus an additional PCMH set of items covering topics beyond the core. The core survey includes multi-item composites assessing access to care, communication with providers, office staff, and an overall provider rating scale. The PCMH item set assesses shared decision making, self-management support, comprehensiveness of care, coordination of care, information about care, and additional aspects of access. All questions assess care in the past 12 months. Complete details of the CAHPS PCMH survey are available at AHRQ [14], including details of the new slightly shortened CAHPS C&G core survey (version 3.0) [12]. Our proposed PCMH survey is not tied to these changes in version 3.0 of the C&G core survey (although items dropped from the C&G version 3.0 are also dropped from our proposed survey).

### 2.2. Sample and Survey Protocol

Practices voluntarily submitting survey data to NCQA must follow procedures that NCQA requires for sample selection. For each survey administered, a random sample of patients is drawn based on the number of clinicians at a practice site (1 clinician in a practice = a required sample size of 128; 2–3 clinicians = 171 sample size; 4–9 clinicians = 343 sample size; 10–13 clinicians = 429 sample size; 14–19 clinicians = 500 sample size; 20–28 clinicians = 643 sample size; 29 or more clinicians = 686 sample size).

Practices choose a random selection of adults (aged ≥18 years) and pediatric (aged <18 years) patients who had at least 1 visit to a provider in the past 12 months prior to survey completion. A parent or guardian is asked to complete the survey for eligible children. In 2013, 268 practices submitted data for adult patients (*n* = 27,896 respondents); 58 practices submitted data for child survey patients (*n* = 4277 respondents). Data were submitted to NCQA in April and September 2013, and surveys had to be administered within the 15 months prior to submission. The last month of data collection allowed was August 2013. The survey administration protocol included mail only, telephone only, mail with telephone follow-up and Internet only administration options. The majority of practices used mail only administration (78% adult, 79% child), with smaller proportions using Internet only (12% adult, 15% child), telephone only (10% adult, 5% child) or mail with telephone follow-up (none in adult, 1% child) administration.

### 2.3. Analysis

We calculated internal consistency reliability (Cronbach’s alpha) of multi-item composites; practice-level unadjusted mean scores for each composite; and site-level reliabilities for each item and composite. Following prior methods used to report CAHPS PCMH results in the literature, we calculated scores using proportional scoring and the summated rating method—*i.e.*, we calculated the mean responses to each item, after transforming each response to a 0–100 scale (100 representing the most positive response on any given item response scale; 0 representing the least positive) [3,7]. For example, on a Yes/No response scale, if “Yes” represents the most positive response, then Yes = 100 and No = 0; on an Always/Usually/Sometimes/Never response scale, if “Always” represents the most positive response, then Always = 100, Usually = 67, Sometimes = 33 and Never = 0. A higher score means that practices were rated more positively for care on that item. We use this 0–100 scale to facilitate comparison of our results to prior, peer-reviewed published CAHPS PCMH results that were reported based on a 0–100 possible range off scores [3,7]. We examined site-level reliabilities by differentiating between-site and within-site variance in one-way ANOVAs [3,7].

We also assessed the extent to which shortening the access and communication composites resulted in changes to the relative ranking of practices. Specifically, we examined the extent to which the ranking of practices shifted under the revised survey composites using two statistical tests. First, we conducted a Pearson’s Chi-Squared test that examined the relationship between (categorical) quintile rankings of practices in the revised *versus* original composites. Second, we examined the rank order correlations among practices using the short and long versions of each composite. Both of these analyses were conducted on each composite (access and communication) for all samples (child and adult).

### 2.4. Proposed Revisions to Shorten the Survey

Based on initial psychometric analysis and stakeholder input, we propose a shorter survey—reducing the adult tool from 52 to 26 items, and the child tool from 66 to 31 items. We consulted 22 stakeholders, representing a variety of perspectives: 11 were clinicians, researchers, survey implementers, those who work with practices to improve patient experiences, and those who use the survey for public reporting purposes; another 11 were patient advocates identified in collaboration with the National Partnership on Women and Families and the Institute for Patient and Family Centered Care. We asked all stakeholders to provide input and select items for a shortened survey based on several key principles: Which items are psychometrically sound (*i.e.*, site-level reliability of 0.70 or higher)? Which items are conceptually central to the PCMH model? Which items are important to consumers? Which items are actionable?

We gathered qualitative input during discussions with stakeholders, including the rationale for prioritizing items based on the above principles. As part of this process, we also asked stakeholders to vote to either “keep” or “drop” items for a shortened survey. The final selection of items was based on this input, including items that were prioritized by stakeholders and garnered the largest number of “keep” votes.

Based on stakeholder input, key changes include reductions in access, communication and comprehensiveness of care composites for the adult and child tool. Because stakeholders did not prioritize the shared decision-making and office staff composites, or several individual (non-composite) items related to access, information, and coordination of care, the proposed shortened survey drops these composites and items (further detail on all items retained for the shortened survey are in the Results).

Item-level results often informed stakeholder input regarding which items could be dropped for a proposed shorter survey. Generally, stakeholders agreed that items achieving estimated reliabilities of less than 0.70 at the practice level could be dropped. For example, an item in the access composite—getting answers to medical questions as soon as needed when phoning one’s provider after-hours—did not achieve 0.70 reliability (0.45 adult, 0.42 child) and was dropped. Self-management support items also did not achieve 0.70 reliability and were dropped.

There were some exceptions, however, including if the item met other guiding principles, such as being conceptually important to the PCMH model or to consumers. For example, a coordination of care item—provider seemed informed and up-to-date about care received from specialists—did not achieve 0.70 reliability (0.66 adult, 0.20 child). However, most stakeholders deemed this item too conceptually important to the PCMH model to be dropped; thus, the item was retained. Conversely, some items achieved 0.70 site-level reliability, but based on concerns over survey length and other guiding principles, stakeholders did not prioritize these items. For example, two items in the access composite (got appointment for routine care; saw provider within 15 min of appointment time) achieved site-level reliabilities above 0.70, but most stakeholders did not deem these two items as conceptually important relative to others in the composite; one of the items also had a lower item-scale correlation with the total composite. Thus, the proposed shortened survey did not include these items.

We sought public comment on the proposed changes in October and November 2014, and received 635 comments—the majority (88%) voted in support the proposed changes [15].

## 3. Results

**Figure 1:**
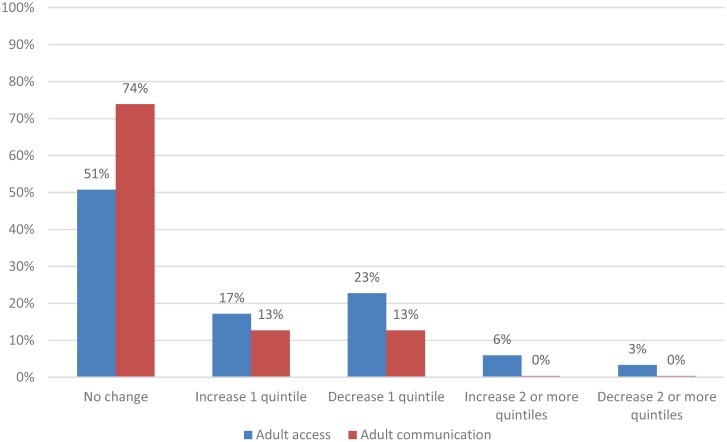
Change in practices’ quintile ranking from original to revised adult composites, CAHPS PCMH 2013 (*n* = 268 practices).

**Figure 2:**
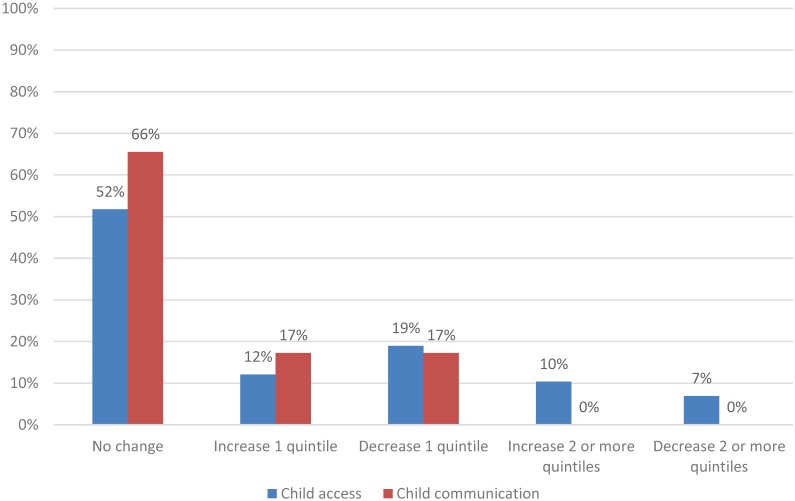
Change in practices’ quintile ranking from original to revised child composites, CAHPS PCMH 2013 (*n* = 58 practices).

**Figure 3:**
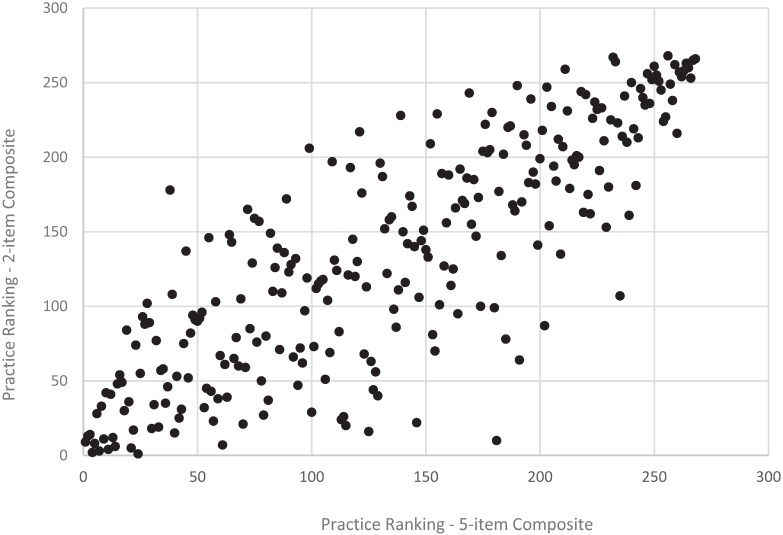
Practice ranking, revised *vs*. original adult access composite, CAHPS PCMH 2013 (*n* = 268 practices).

**Figure 4:**
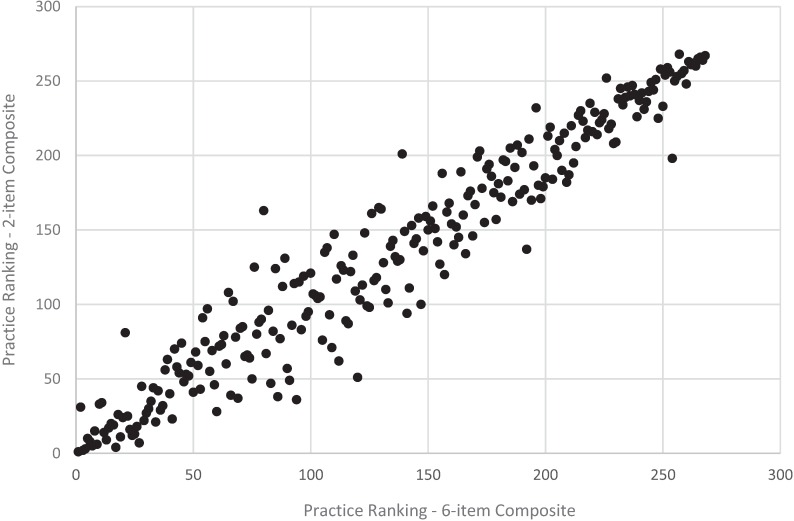
Practice ranking, revised *vs*. original adult communication composite, CAHPS PCMH 2013 (*n* = 268 practices).

**Figure 5:**
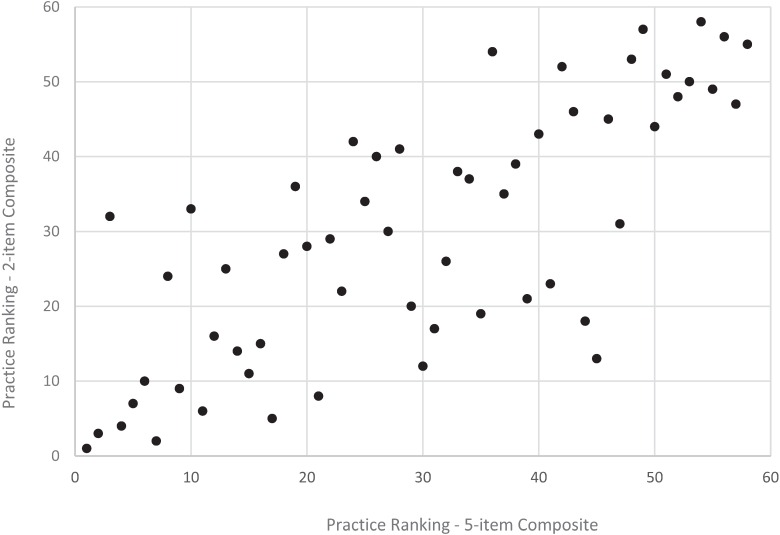
Practice ranking, revised *vs*. original child access composite, CAHPS PCMH 2013 (*n* = 58 practices).

**Figure 6:**
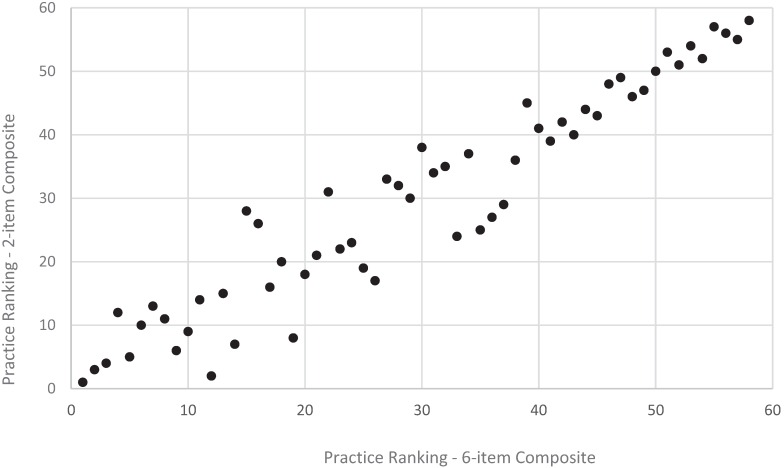
Practice ranking, revised *vs*. original child communication composite, CAHPS PCMH 2013 (*n* = 58 practices).

A total of 268 practices submitted data on the adult survey and 58 practices submitted data on the child survey. The mean number of respondents per practice was 104 for the adult survey and 74 for the child survey. The overall response rate was 27% for adults and 23% for children. Respondent characteristics are presented in Table 1. For the adult survey, the majority of respondents were female (61%) and aged 55–64 years (25%). Most self-rated their general health as good (36%) and their mental health as very good (35%). For the child survey (filled out by the child’s parent or guardian), the majority of respondents were also female (89%). Parental ratings of child health on the child survey were better overall than self-rated health on the adult survey, with excellent general and mental health ratings of 57% and 56%, respectively, for the child sample. The majority of practices, for both adult and child samples, were comprised of multiple providers (four or more), with ownership under a hospital, health system, or health plan (rather than physician owned) and located in the Northeast census region. Below we describe key results for the current PCMH composites and items for both adults (Table 2) and children (Table 3), as well as the impact of shortening the survey (Table 4 and Figure 1, Figure 2, Figure 3, Figure 4, Figure 5 and Figure 6). Table 2 and Table 3 indicate, in italics, all items retained for the shortened survey.

**Table 1:** Characteristics of adult survey respondents and children who were the patients asked about in child surveys, CAHPS PCMH ^1^, 2013.

Characteristic	Category	Respondents for Adult Survey, *n* = 268 Practices (27,896 Individuals)		Children Asked about, *n* = 58 Practices (4277 Individuals)	
		*N*	Percent	*N*	Percent
Age	<18 years	0	**0**	3341	**99%**
	18–24 years	947	**4%**	26	**1%**
	25–34 years	1936	**7%**	-	-
	35–44 years	2540	**9%**	-	-
	45–54 years	4808	**18%**	-	-
	55–64 years	6764	**25%**	-	-
	65–74 years	5823	**21%**	-	-
	≥75 years	4550	**17%**	-	-
Gender	Male	10,682	**39%**	435	**11%**
	Female	16,697	**61%**	3683	**89%**
Ethnicity	Hispanic	1729	**7%**	318	**8%**
	Not Hispanic	24,685	**94%**	3783	**92%**
General Health	Excellent	3638	**13%**	2393	**57%**
	Very Good	9485	**35%**	1383	**33%**
	Good	9773	**36%**	340	**8%**
	Fair	3761	**14%**	51	**1%**
	Poor	717	**3%**	6	**0%**
Mental Health	Excellent	6990	**26%**	2319	**56%**
	Very Good	9533	**35%**	1217	**29%**
	Good	7489	**27%**	485	**12%**
	Fair	2752	**10%**	135	**3%**
	Poor	545	**2%**	19	**1%**
No. of visits past year	1	6147	**23%**	1221	**30%**
	2	7269	**27%**	1151	**28%**
	3	5038	**19%**	760	**18%**
	4	3880	**15%**	434	**11%**
	5–9	3325	**13%**	500	**12%**
	≥10	981	**4%**	64	**2%**

^1^ CAHPS PCMH = Consumer Assessment of Healthcare Providers and Systems—Patient-Centered Medical Home.

### 3.1. Internal Consistency Reliabilities

The majority of multi-item composites formed an internally consistent scale in current versions of both adult (Table 2) and child surveys (Table 3)—with four composites meeting the recommended standard of a 0.70 or higher Cronbach’s α: communication with providers, six items (Cronbach’s α = 0.92 adult and 0.91 child); office staff interaction, two items (0.84 adult and 0.85 child); access to care, five items (0.81 adult; 0.70 child); and comprehensiveness of behavioral care, three items (0.79 adult-only composite). Only two composites did not achieve the 0.70 level: self-management support (0.66 adult; 0.60 child) and shared decision making (0.65 adult-only composite).

**Table 2:** 2013 results for the adult CAHPS PCMH Survey (*n* = 268 practices). Items in italics recommended for revised survey.

Item	Item #	Core or PCMH ^1^	Response Set ^2^	Correlation with Total (Composite) ^3^		Composite		Practice Level Reliability ^5^
				With Original Composite	With Revised Composite	Mean ^4^	SD	
***Rating of provider***	32	Core	0–10	-	-	89.47	4.20	0.80
**Access (5 items original, 2 items revised)**						**76.02**	**9.69**	**0.94**
*Got appointment for urgent care as soon as needed*	6	Core	N-A	**0.74**	**0.74**	82.97	9.34	0.84
Got appointment for check-up or routine care as soon as needed	9	Core	N-A	**0.76**	-	85.20	7.15	0.87
*Got answer to medical question the same day you phoned*	14	Core	N-A	**0.68**	**0.74**	79.24	8.96	0.75
Got answer to medical question as soon as you needed when phoned after hours	16	Core	N-A	**0.33**	-	73.31	19.05	0.45
Saw provider within 15 min of appointment time	18	Core	N-A	**0.57**	-	65.56	14.79	0.95
**Items not scored in composite**								
Days you had to wait for an appointment for urgent care	7	PCMH	0–7 Days	-	-	25.52	12.65	0.90
Got needed care on evenings, weekends, or holidays	12	PCMH	N-A	-	-	48.76	19.99	0.67
**Information (2 items, not scored as a composite)**								
*Got information about what to do if you needed care on evenings, weekends, or holidays*	10	PCMH	Y-N^	-	-	70.64	10.56	0.78
Received reminders between visits	17	PCMH	Y-N	-	-	66.25	11.83	0.79
**Communication (6 items original, 2 items revised)**						**91.24**	**4.15**	**0.82**
*Provider explained things in a way that was easy to understand*	19	Core	N-A	**0.92**	**0.79**	92.68	4.00	0.78
Provider listened carefully	20	Core	N-A	**0..93**	-	92.33	3.96	0.73
Provider gave easy to understand instructions about taking care of health problems or concerns	22	Core	N-A	**0.92**	-	91.23	4.38	0.71
*Provider seemed to know important information about your medical history*	23	Core	N-A	**0.83**	**0.79**	87.53	5.61	0.81
Provider respected what you had to say	24	Core	N-A	**0.91**	-	93.85	3.78	0.74
Provider spent enough time with you	25	Core	N-A	**0.87**	-	90.41	4.85	0.80
**Coordination of Care (3 items, not scored as a composite)**								
*Provider’s office followed up to give you results of blood test, X-ray, or other test*	27	Core	N-A	-	-	82.46	9.08	0.85
*Provider seemed informed and up-to-date about care you got from specialists*	34	PCMH	N-A	-	-	79.53	7.32	0.66
*Talked with you about your prescriptions*	38	PCMH	N-A	-	-	84.56	9.50	0.82
**Comprehensiveness-Behavioral/whole person (3 items original, single item revised)**						**43.82 **	**13.23 **	**0.91 **
Talked about personal or family problem/alcohol or drug use	39	PCMH	Y-N	**0.86**	-	45.94	16.11	0.92
*Talked about worry and stress in your life*	40	PCMH	Y-N	**0.89**	-	49.35	14.33	0.89
Talked about feeling sad or depressed	41	PCMH	Y-N	**0.87**	-	35.98	11.74	0.83
**Self-Management Support (2 items original & revised)**						**45.45**	**10.52**	**0.81**
*Work with you to set specific goals for your health*	35	PCMH	Y-N	**0.78**	**0.78**	56.09	11.81	0.78
*Ask if there are things make it hard to take care of your health*	36	PCMH	Y-N	**0.78**	**0.78**	34.53	10.57	0.75
**Shared Decision Making (3 items)**						**79.51**	**6.46**	**0.58**
Provider talked about reasons to take a medicine	29	PCMH	Not-A lot	**0.68**	-	85.56	4.62	0.39
Provider talked about reasons not to take a medicine	30	PCMH	Not-A lot	**0.73**	-	71.42	7.96	0.49
Provider asked what you thought was best for you regarding medicine	31	PCMH	Y-N	**0.65**	-	81.56	9.66	0.52
**Office Staff (2 items)**						**87.43**	**5.78**	**0.87**
Office staff at this office were as helpful as you though they should be	42	Core	N-A	**0.92**	-	84.09	6.68	0.86
Office staff at this office treated you with courtesy and respect	43	Core	N-A	**0.92**	-	90.82	5.12	0.84

CAHPS PCMH = Consumer Assessment of Healthcare Providers and Systems—Patient-Centered Medical Home; Item # refers to the numbering of the survey item within the survey instrument; SD = standard deviation; ^1^ Indicates whether the item is part of the Clinician and Group-CAHPS core survey or an item newly developed for the PCMH survey; ^2^ Indicates the response sets used for the item: N-A = never, sometimes, usually, always, Y-N = yes, no, Not-A lot = Not at all, A little, Some, A lot; ^3^ Item-scale correlation is corrected for the overlap of the item with the scale (composite) score; ^4^ Mean scores are derived by averaging responses that have been rescaled to a 0–100 range, where 100 represents the most positive response. For example, on a Yes/No response scale, if “Yes” represents the most positive response, then Yes = 100 and No = 0; on an Always/Usually/Sometimes/Never response scale, if “Always” represents the most positive response, then Always = 100, Usually = 67, Sometime = 33 and Never = 0. A higher score means that practices were rated more positively for care on that item; ^5^ See methods for explanation.

**Table 3:** 2013 results for the child CAHPS PCMH Survey (*n* = 58 practices). Items in italics recommended for revised survey.

Item	Item #	Core or PCMH ^1^	Response Set ^2^	Correlation with Total (Composite) ^3^		Composite		Practice Level Reliability ^5^
				With Original Composite	With Revised Composite	Mean ^4^	SD	
***Rating of provider***	35	Core	0–10			89.47	4.21	0.80
**Access (5 items original, 2 items revised)**						**80.53**	**7.29**	**0.88**
*Got appointment for urgent care as soon as needed*	13	Core	N-A	**0.61**	**0.61**	90.06	5.62	0.69
Got appointment for check-up or routine care as soon as needed	16	Core	N-A	**0.51**	--	87.02	6.10	0.75
*Got answer to medical question the same day you phoned provider’s office*	21	Core	N-A	**0.54**	**0.61**	90.18	5.99	0.70
Got answer to medical question as soon as you needed when phoned provider’s office after hours	23	Core	N-A	**0.40**	--	87.41	10.47	0.42
Saw provider within 15 min of appointment time	25	Core	N-A	**0.60**	--	67.64	11.59	0.88
**Items not scored in composite**								
Days you had to wait for an appointment for urgent care	14	PCMH	0–7 Days	--	--	91.42	6.45	0.83
Got needed care on evenings, weekends, or holidays	19	PCMH	N-A	--	--	69.37	15.71	0.71
**Information (2 items, not scored as a composite)**								
*Got information about what to do if you needed care on evenings, weekends, or holidays*	17	PCMH	Y-N	--	--	81.02	9.08	0.72
Received reminders between visits	24	PCMH	Y-N	--	--	58.24	15.00	0.85
**Communication (6 items original, 2 items revised)**						**93.29**	**3.78**	**0.70**
*Provider explained things in a way that was easy to understand*	26	Core	N-A	**0.89**	**0.86**	94.94	3.69	0.66
Provider listened carefully	27	Core	N-A	**0.93**	--	94.41	3.22	0.51
Provider gave easy to understand instructions about taking care of health problems or concerns	29	Core	N-A	**0.77**	--	94.00	3.82	0.52
*Provider seemed to know important information about your medical history*	30	Core	N-A	**0.90**	**0.86**	89.88	5.83	0.74
Provider respected what you had to say	31	Core	N-A	**0.92**	--	94.96	3.10	0.49
Provider spent enough time with you	32	Core	N-A	**0.89**	--	92.54	4.01	0.58
**Coordination of care (3 items, not scored as composite)**								
*Provider’s office followed up to give you results of blood test, X-ray, or other test*	*34*	Core	N-A	--	--	84.88	9.80	0.48
*Provider seemed informed and up-to-date about care you got from specialists*	*37*	PCMH	N-A	--	--	77.59	10.69	0.20
Talked with you about your prescriptions	52	PCMH	N-A	--	--	87.63	8.75	0.5
**Comprehensiveness-Child Development (6 items original, single item revised)**						**61.79**	**12.08**	**0.87**
Talked about child’s learning ability	38	Core	Y-N	0.85	-	47.11	12.58	0.73
Talked about behaviors that are normal for child at this age	39	Core	Y-N	0.85	-	68.79	14.06	0.83
Talked about how your child’s body is growing	40	Core	Y-N	0.62	-	79.67	12.26	0.81
Talked about child’s moods and emotions	41	Core	Y-N	0.87	-	60.09	14.24	0.81
Talked about how much time child spends in front of a computer/TV	44	PCMH	Y-N	0.78	-	49.96	19.26	0.91
Talked about how child gets along with others	47	Core	Y-N	0.78	-	53.39	16.26	0.85
**Comprehensiveness-Child Prevention (5 items original, 2 items revised)**						**58.84**	**13.76**	**0.88**
Talked about things to do to keep child from getting injured	42	Core	Y-N	**0.82**	-	58.14	14.49	0.81
Given information about keeping child from getting injured	43	Core	Y-N	**0.77**	-	51.45	16.35	0.83
Talked about food your child eats	45	Core	Y-N	**0.82**	-	78.68	13.89	0.85
*Talked about exercise your child gets*	46	Core	Y-N	**0.75**	**0.62**	67.20	13.55	0.80
*Talked about if there were problems in household that might affect child*	48	Core	Y-N	**0.73**	**0.62**	46.18	18.20	0.88
**Self-Management Support (2 items original & revised)**						**33.43**	**10.65**	**0.72**
*Work with you to set specific goals for your health*	*49*	PCMH	Y-N	**0.82**	-	45.43	13.13	0.68
*Ask you if there are things that make it hard for you to take care of your health*	*50*	PCMH	Y-N	**0.82**	-	21.29	9.14	0.65
**Office Staff (2 items)**						**87.43**	**5.67**	**0.84**
Office Staff at this office were as helpful as you though they should be	53	Core	N-A	**0.90**	-	84.92	6.36	0.82
Office Staffs at this office treated you with courtesy and respect	54	Core	N-A	**0.90**	-	89.93	5.29	0.82

CAHPS PCMH = Consumer Assessment of Healthcare Providers and Systems—Patient-Centered Medical Home; Item # refers to the numbering of the survey item within the survey instrument; SD = standard deviation; ^1^ Indicates whether the item is part of the Clinician & Group-CAHPS core survey or an item newly developed for the PCMH survey; ^2^ Indicates the response sets used for the item: N-A = never, sometimes, usually, always, Y-N = yes, no, Not-A lot = Not at all, A little, Some, A lot; ^3^ Item-scale correlation is corrected for the overlap of the item with the scale (composite) score; ^4^ mean scores are derived by averaging responses that have been rescaled to a 0–100 range, where 100 represents the most positive response. For example, on a Yes/No response scale, if “Yes” represents the most positive response, then Yes = 100 and No = 0; on an Always/Usually/Sometimes/Never response scale, if “Always” represents the most positive response, then Always = 100, Usually = 67, Sometime = 33 and Never = 0. A higher score means that practices were rated more positively for care on that item; ^5^ see methods for explanation.

**Table 4:** Comparison of original composites and revised composites: Adult (*n* = 268 practices) and child (*n* = 58 practices) CAHPS PCMH Survey, 2013. (*Items in italics recommended for revised survey*).

Item	# of Items	Mean	SD	Internal Consistency Reliability ^1^	Practice Level Reliability	Number of Responses per Practice Needed to Achieve 0.70, 0.80, and 0.90 Reliability ^2^		
						Reliability = 0.70	Reliability = 0.80	Reliability = 0.90
**Adult Survey**								
Original Access	5	76.02	9.69	0.81	0.94	**14**	**24**	**55**
*Revised Access*	2	81.50	8.41	0.67	0.85	**26**	**45**	**101**
Original Communication	6	91.24	4.15	0.92	0.82	**52**	**89**	**200**
*Revised Communication*	2	90.11	4.55	0.72	0.82	**50**	**85**	**191**
Shared Decision Making	3	79.51	6.46	0.65	0.58	**84**	**144**	**324**
Self-Management Support	2	45.45	10.52	0.66	0.81	**55**	**94**	**211**
Comprehensiveness-Behavioral	3	43.82	13.23	0.79	0.91	**21**	**37**	**83**
*Revised Comprehensiveness-Adult Behavioral* (*Single item: Talked about worry and stress in your life*)	1	49.35	14.33	NA	0.89	**29**	**50**	**113**
Office Staff	2	87.43	5.78	0.84	0.87	**35**	**59**	**134**
**Child Survey**								
Original Access	5	80.53	7.29	0.70	0.88	**22**	**37**	**84**
*Revised Access*	2	90.03	5.27	0.56	0.77	**36**	**62**	**139**
Original Communication	6	93.29	3.78	0.91	0.70	**71**	**121**	**272**
*Revised Communication*	2	92.40	4.54	0.68	0.75	**55**	**94**	**211**
Comprehensiveness-Child Development	6	61.79	12.08	0.81	0.87	**18**	**32**	**71**
*Revised Comprehensiveness-Child Development* (*Single item: Talked about behaviors that are normal for child at this age*)	1	68.79	14.06	NA	0.83	**34**	**58**	**129**
Comprehensiveness-Child Prevention	5	58.84	13.76	0.81	0.88	**17**	**30**	**67**
*Revised Comprehensiveness-Child Prevention*	2	56.65	14.35	0.59	0.88	**21**	**37**	**83**
Self-Management Support	2	33.43	10.65	0.60	0.72	**61**	**104**	**234**
Office Staff	2	87.43	5.67	0.85	0.84	**31**	**53**	**120**

CAHPS PCMH = Consumer Assessment of Healthcare Providers and Systems—Patient-Centered Medical Home; Item # refers to the numbering of the survey item within the survey instrument; SD = standard deviation; ^1^ based on Cronbach’s Alpha. See methods for explanation; ^2^ the estimated number of responses are based on the Spearman-Brown formula.

Reducing the number of items in existing composites generally led to reductions in internal consistency reliability (Table 4). For the access composite for adults, reducing from five to two items led to reduction in internal consistency reliability from 0.81 to 0.67. For the communication composite in adults, reducing from six to two items changed the internal consistency reliability from 0.92 to 0.72. These findings are to be expected since the Cronbach’s alpha increases as the number of items in a scale increases. Only the internal consistency of the access item for adults (0.67) fell below the recommended level of 0.70.

These patterns were also generally found in the child results (Table 4). However, three child composites fell below the recommended internal consistency level of 0.70 when revised: access (0.56 for the two-item scale), communication (0.68 for the two-item scale), and comprehensiveness in child prevention (0.59 for the two-item scale).

### 3.2. Practice Level Reliabilities

Practice-level reliabilities achieved the recommended level of 0.70 or higher for most current versions of multi-item composites in the adult (Table 2) and child (Table 3) surveys, with the exception of the shared decision making composite (0.58).

Reducing the number of items in existing composites led to reductions in practice level reliability for only the access composite (Table 4). Reducing the access composite from five to two items led to reduction in practice level reliability from 0.94 to 0.85 for adults, and 0.88 to 0.77 for children. There was no reduction in practice level reliability for the communication composite in adults or children (e.g., 0.82 for both the six-item and two-item scale in adults), nor for the child comprehensiveness of preventive care composite (0.88 for both the five-item and two-item scale).

### 3.3. Item to Scale Correlations and Unadjusted Mean Scores

Item-scale correlations (Table 2 and Table 3) provided support for the reduced composites (all correlations achieving levels of 0.50 or higher, and correlating about as highly as many items in the original), with some exceptions. For the adult communication and the child comprehensiveness of preventive care composite, items in the reduced two-item composite correlated more weakly than the lowest correlation in the original multi-item composites (e.g., for adult communication: 0.79 for the two-item scale and 0.83 for the weakest correlation in the six-item scale; for child comprehensiveness of preventive care: 0.62 for the two-item scale and 0.73 for the weakest correlation in the five-item scale). However, these item-scale correlations still achieved 0.50. We also note that the correlations for two-item scales should not be interpreted as correlations with a true “scale” as they relate one item to only one other item.

Unadjusted mean scores were also generally stable with the reductions; the largest difference being a five-point score improvement for the adult access composite (76.0 for the original six-item scale to 81.5 for the short two-item scale).

### 3.4. Responses Estimated to Obtain Site-level Reliabilities of 0.70, 0.80, 0.90

The number of estimated responses per practice needed to achieve reliabilities of 0.70, 0.80, and 0.90 are presented in Table 4. For the adult survey, the number of responses for a reliability of 0.70 ranged from 14 to 84, with a higher minimum number of responses needed to achieve the same reliability in the revised composites (minimum of 26, maximum of 50). For the child survey, the number of responses for a reliability of 0.70 ranged from 17 to 71, with a higher minimum number of responses needed to achieve the same reliability in the revised composites (minimum of 21, maximum of 55).

### 3.5. Relative Ranking of Practices under Revised Survey Composites

Shortening both the adult and child composites for access and communication resulted in more changes in the relative ranking of practices for the access composite compared to the communication composite. Specifically, for the communication composite, results from the quintile analysis showed that 74% of adult practices did not change rank while 25% changed one quintile rank (Figure 1); 66% of child practices did not change quintile rank while 35% changed one quintile rank (Figure 2). For the access composite, however, there were more changes based on quintile ranks: 51% of adult practices did not change rank while 40% changed one quintile rank (Figure 1); 52% of child practices did not change rank while 31% changed one quintile rank (Figure 2).

Results from the (full) rank order correlation analysis were consistent with results from the quintile ranking analysis. Specifically, for the communication composite, long and short versions of the composite resulted in similar practice rankings for both the adult (*r* = 0.97, *p* < 0.001) and child (*r* = 0.96, *p* < 0.001) versions of the composites (Figure 4 and Figure 6). For the access composite, long and short versions of the composite also resulted in more changes in rankings for the adult (*r* = 0.83, *p* < 0.001) and child (*r* = 0.76, *p* < 0.001) composites (Figure 3 and Figure 5) compared to the communication composite—although these still meet common recommended levels of 0.70 or higher for a strong, positive correlation.

## 4. Discussion

This study provides further support for the reliability and validity of the current CAHPS PCMH survey, based on updated data across a larger sample, and characterizes the psychometric impact of shortening the survey. Importantly, our findings suggest that a shorter adult survey is possible. Unadjusted mean scores were also generally stable with the reduction; the largest difference being a five-point score improvement for the adult access composite. For both the adult and child surveys, the reductions did result in more changes in the relative ranking of practices for the access composite, compared to the communication composite.

In general, internal consistency reliability for the multi-item composites exceeded or equaled original published field test results [3]. Estimates of site-level reliability also indicate that a reliability of 0.70 or higher can generally be achieved for most multi-item composites. However, borderline site-level reliability among select composites and items suggest that, despite their salience to the PCMH care model, these items and composites may be considered for removal to streamline the survey and its effectiveness and uptake. Previous research by the CAHPS Consortium suggests that survey length generally does not affect survey response rates, with prior findings suggesting that the number of survey questions that respondents were required to answer, from as few as 23 to as many as 95, had little effect on response rates and respondents were as likely to answer a relatively longer survey as a shorter one [16]. However, recent input from a diverse group of stakeholders under NCQA’s PCMH recognition program have suggested a need to consider shortening the survey in order to increase response rates. Both NCQA and the CAHPS Consortium have conducted research to re-evaluate the PCMH survey, and, as of the time of this present study, have each put forth their own proposals for changes to the survey [11,15], with the CAHPS Consortium finalizing their revised version of the CAHPS Clinician and Group survey (version 3.0) in July 2015, reducing the length from 34 to 31 items [12]. Additional possibilities for shortening the CAHPS Clinician and Group survey have also since been published [13].

The results here suggest that reduction in length are possible; despite some reduction in psychometric properties, the reduced adult survey would still generally meet standard definitions of a psychometrically sound survey; however, given three child composites fell below the recommended internal consistency level of 0.70 when revised, further testing is recommended to establish appropriate criteria for shortening the child survey. For example, further work could investigate whether internal consistency reliability suffered because these composites may not have reflected “true” scales, whether the smaller child survey samples may have influenced practice-level reliability—which in turn influenced item-level results and decisions to drop item, or whether there may be something else altogether beyond these psychometric concerns—such as the possibility of more variability in the kind of care pediatric populations require.

Additionally, while a shorter survey addresses ongoing concerns about survey length, further work should also investigate related issues of survey response and uptake, including whether a shorter survey facilitates meaningful improvement in response rates, or facilitates opportunities for customization of the survey to fit practice needs. Input from consumers and families about the relevance of these measures for decision-making as well as practice input on the usefulness in quality improvement are also key considerations.

In recommending any further potential changes to shorten the survey, several overarching principles should be taken into account, including some of those used in the current study. First, any reduction needs to be weighed not only against its impact on psychometric attributes, but also against goals for survey use. During stakeholder discussions and public comment fielding, many indicated the importance of having a shorter survey that meets a mix of both accountability and quality improvement needs. One useful principle already used in the current study is to consider whether an item is actionable, which speaks to its usefulness from both an accountability and quality improvement perspective.

Second, reductions may need to be considered for only certain composites *versus* all composites. In the current study, some reductions achieved higher internal consistency reliability than others, begging the question of whether a broad approach of shortening all composites is too “blunt”, and if reductions should instead be customized to only some portions of the survey.

Finally, relevant to these concept of customization, any survey change needs to consider the increasing attention towards flexibility. Although there was overwhelming support for shortening the survey, there were also diverse opinions regarding which items should be dropped. Given the CAHPS Consortium, NCQA and other groups (including the Massachusetts Health Quality Partners) have each proposed slightly different approaches for shortening the survey, this begs the further question of whether the route to a shorter survey should emphasize not so much the selection of specific items, but rather the creation of a flexible route to assessment. The literature has already begun to acknowledge the need to strike this balance, calling for patient surveys, such as the CAHPS surveys, to allow for variation, while retaining common core elements as a “foundation” to facilitate alignment and standardization [17].

This study had some limitations. First, response rates were lower than seen in some other surveys, although they are similar to response rates in some implementations of CAHPS surveys [3]. While a low response rate may not have affected the psychometric results presented in this study, this is an important limitation. As we were unable to examine differences between non-responders and responders, the study results must be interpreted with caution and may not be generalizable. Second, the majority of practices were from the northeast area, which also affects the generalizability of our results. However, unlike prior published findings of the CAHPS PCMH survey, practices from most major census regions (west, midwest, northeast), except the south, submitted data. Despite these limitations, this study provides important information on the psychometric impact of shortening the survey, and opens up possibilities for assessing patient experiences in medical home settings where survey length or burden may be a concern.

As PCMH adoption expands, the ability to evaluate the PCMH promise of improving patient experiences and other aspects of care remains essential. The current literature acknowledges that more evidence is generally needed to determine the effects of the PCMH on select outcomes [2]. Given the concerns around survey length, opportunities to shorten the CAHPS PCMH survey would complement current measurement efforts to evaluate PCMH settings. Further research should address and further delineate the approaches needed to ensure that the CAHPS PCMH plays a useful role in optimizing patient experiences in PCMH and other efforts to reform the health system, whether it is investigating approaches to improving survey response or uptake, the relevance of survey items and composites to inform quality improvement, or the incorporation of new methods to efficiently assess priority domains, while retaining opportunities for shortening and customizing the survey.

## 5. Conclusions

In conclusion, the current study provided an opportunity to evaluate key aspects of the PCMH model of care across a large group of medical practices. The findings show that shortening the survey—in response to survey length concerns—reduces reliability, but still meets general definitions of a sound survey for the adult version; however, further testing is recommended to establish appropriate criteria for shortening the child survey. Future opportunities to evaluate PCMH patient experiences, and to improve current measures for doing so, remain key towards assessing whether the PCMH translates into improvements for patients.
